# 3D Dixon water-fat LGE imaging with image navigator and compressed sensing in cardiac MRI

**DOI:** 10.1007/s00330-020-07517-x

**Published:** 2020-12-02

**Authors:** Martin Georg Zeilinger, Marco Wiesmüller, Christoph Forman, Michaela Schmidt, Camila Munoz, Davide Piccini, Karl-Philipp Kunze, Radhouene Neji, René Michael Botnar, Claudia Prieto, Michael Uder, Matthias May, Wolfgang Wuest

**Affiliations:** 1 Institute of Diagnostic Radiology, University Hospital of Erlangen, Erlangen, Germany; 2 Cardiovascular MR Predevelopment, Siemens Healthcare GmbH, Erlangen, Germany; 3 School of Biomedical Engineering and Imaging Sciences, King’s College London, London, UK; 4 Advanced Clinical Imaging Technology, Siemens Healthcare IM BM PI, Lausanne, Switzerland; 5 MR Research Collaborations, Siemens Healthcare GmbH, Frimley, UK

**Keywords:** Three-dimensional imaging, Cardiac, Magnetic resonance imaging, Myocardium, Pericardium

## Abstract

**Objectives:**

To evaluate an image-navigated isotropic high-resolution 3D late gadolinium enhancement (LGE) prototype sequence with compressed sensing and Dixon water-fat separation in a clinical routine setting.

**Material and methods:**

Forty consecutive patients scheduled for cardiac MRI were enrolled prospectively and examined with 1.5 T MRI. Overall subjective image quality, LGE pattern and extent, diagnostic confidence for detection of LGE, and scan time were evaluated and compared to standard 2D LGE imaging. Robustness of Dixon fat suppression was evaluated for 3D Dixon LGE imaging. For statistical analysis, the non-parametric Wilcoxon rank sum test was performed.

**Results:**

LGE was rated as ischemic in 9 patients and non-ischemic in 11 patients while it was absent in 20 patients. Image quality and diagnostic confidence were comparable between both techniques (*p* = 0.67 and *p* = 0.66, respectively). LGE extent with respect to segmental or transmural myocardial enhancement was identical between 2D and 3D (water-only and in-phase). LGE size was comparable (3D 8.4 ± 7.2 g, 2D 8.7 ± 7.3 g, *p* = 0.19). Good or excellent fat suppression was achieved in 93% of the 3D LGE datasets. In 6 patients with pericarditis, the 3D sequence with Dixon fat suppression allowed for a better detection of pericardial LGE. Scan duration was significantly longer for 3D imaging (2D median 9:32 min vs. 3D median 10:46 min, *p* = 0.001).

**Conclusion:**

The 3D LGE sequence provides comparable LGE detection compared to 2D imaging and seems to be superior in evaluating the extent of pericardial involvement in patients suspected with pericarditis due to the robust Dixon fat suppression.

**Key Points:**

*• Three-dimensional LGE imaging provides high-resolution detection of myocardial scarring.*

*• Robust Dixon water-fat separation aids in the assessment of pericardial disease.*

*• The 2D image navigator technique enables 100% respiratory scan efficacy and permits predictable scan times.*

## Introduction

In cardiac MRI (CMR), late gadolinium enhancement (LGE) is an important tool in the assessment of necrosis and fibrosis after myocardial infarction, as well as in various non-ischemic cardiomyopathies, e.g., to determine the severity of infectious myocarditis [1]. The concept is based on a delayed hyperenhancement due to increased extracellular myocardial volume in the aforementioned diseases [2]. A two-dimensional (2D) inversion recovery fast spoiled gradient-echo or balanced steady-state free precession (bSSFP) sequence acquired in multiple breath-holds is commonly used to evaluate LGE. Such 2D approaches may suffer from slice misregistration (incomplete breath-holding), artifacts due to respiratory motion, and constraints in spatial resolution [3]. Alternatively, 3D imaging has been proposed with the potential advantage of isotropic resolution and extended myocardial coverage, allowing for assessment of scar tissue in thinner myocardial walls like the atria or the right ventricle [4–6]. Previously reported 3D LGE sequences, however, suffered from long breath-hold duration > 20 s [7], thereby compromising the efficiency in potentially vulnerable patients [8]. In order to acquire images under free breathing, several compensation techniques have been published to minimize respiratory-induced motion artifacts [9–11]. Recently developed 2D image navigation (iNAV) provides direct respiratory motion tracking of the heart in head–foot and left–right directions [12]. This technique outperforms conventional respiratory motion compensation techniques such as diaphragmatic navigator gating [9], because it does not require a motion model and enables 100% respiratory scan efficiency resulting in shorter and predictable scan time, and has been successfully tested in 3D LGE imaging with lower acquired resolution of (2.0 mm)^3^ [10, 13]. In order to further accelerate 3D LGE image acquisition, a combination of parallel imaging and compressed sensing was proposed [5]; however, this approach uses diaphragmatic navigator gating. Compressed sensing is based on the three principles of a sparse representation of the acquired object, a pseudo-random sub-sampling of *k*-space in order to create incoherent (noise-like) undersampling artifacts and a non-linear, iterative image reconstruction [14–17].

Besides reducing motion artifacts and scan time, the diagnostic value of LGE imaging can be further improved with fat suppression. Missing or insufficient fat suppression may lead to an inadequate discrimination e.g., between epicardial fat, myocardial fibrosis, and enhancing pericardium in pericarditis or between intramyocardial fat deposition and fibrosis in chronic myocardial infarction [18]. Both conventional fat-selective saturation pulses [19] and chemical shift–based water-fat separation (Dixon) have been proposed in LGE imaging [20]. Fat saturation is limited by high sensitivity to magnetic field inhomogeneity resulting in imperfect suppression, edge enhancement, and mottled blood pool appearance [21]. In this regard, Dixon LGE outperformed fat-saturated 3D LGE [21]. Furthermore, the Dixon technique provides additional information since in-phase and water-only images are available separately.

The purpose of this study was to compare a novel iNAV-based, isotropic high-resolution 3D LGE prototype sequence with compressed sensing and Dixon water-fat separation to our standard 2D LGE sequence without fat suppression in a clinical routine setting.

## Materials and methods

### Patients

Consecutive patients scheduled for CMR including LGE were screened for study participation, and a total of 40 patients were prospectively included. Indications for MRI were detection of scar tissue after infarction (*n* = 11), pericarditis or myocarditis (*n* = 20), and various cardiomyopathies such as non-compaction cardiomyopathy, arrhythmogenic right ventricular dysplasia (ARVD), amyloidosis, systemic lupus erythematosus, or sarcoidosis (*n* = 9). Exclusion criteria for this study were presence of any contraindications for MRI, i.e., known allergies to contrast material, pregnancy, and impaired renal function (estimated glomerular filtration rate below 30 ml/min), as well as age younger than 18 years, critically ill patients, and permanent arrhythmia. All patients signed informed consent. The study protocol was approved by the local institutional review board and is compliant with the Health Insurance Portability and Accountability Act (HIPAA) criteria.

### CMR protocol

CMR imaging was performed on a 1.5-T MRI system (MAGNETOM Aera, Siemens Healthcare) with dedicated phased-array cardiac receiver coils (18-channel body coil, 32-channel spine coil). The image acquisition followed standardized protocols [22]. Cine images in three long-axis (4,3,2 chamber view) and contiguous short-axis views covering the entire left ventricle from the base to the apex were acquired using a bSSFP sequence. For LGE imaging, an intravenous contrast bolus of 0.2 mmol/kg gadobutrol (Gadovist, Bayer Inc.) was administered according to current recommendations [22]. Two separate LGE sequences were performed: (1) high-resolution (1.3 mm)^3^ isotropic iNAV-3D LGE with compressed sensing, Dixon-based fat suppression separation, and inversion pulses every RR interval and (2) the institutional standard 2D LGE sequence using T1w fast gradient-echo phase-sensitive inversion recovery (PSIR) sequences with inversion pulses every other RR interval. The optimal inversion time to null the left ventricular (LV) myocardium was determined individually prior to each LGE sequence using a Look-Locker sequence. To avoid bias between the different LGE acquisition time points, the total study group of 40 patients was divided into two. Twenty patients were randomly assigned to undergo the 3D acquisition prior to 2D (group 1) and the other 20 patients vice versa (group 2).

### Two-dimensional LGE sequence

A segmented 2D PSIR gradient-echo pulse sequence with identical slice positioning to cine bSSFP images was performed in breath-hold according to current recommendations [22, 23]. A short-axis (SAX) stack covering the ventricles was acquired with the following parameters: in-plane resolution of 1.4 × 1.4 mm, slice thickness of 8 mm, interslice gap of 2 mm, TR of 8.35 ms, TE of 3.23 ms, flip angle of 25°, matrix of 156 × 256, FOV of 276 × 340 mm (no phase oversampling but reconstruction of only 81.3% of the 340-mm-phase FOV, i.e., 276 mm), and number of averages of 1.

Long-axis (LAX) planes in 4-chamber view (horizontal long axis), 3-chamber view (left ventricular outflow view), and 2-chamber view (vertical long axis) were acquired with the same parameters. The inversion time was determined by an inversion time Look-Locker scout sequence performed immediately before the acquisition of the first slice of the short axis stack, and typically ranged around 254 ± 51 ms.

Acquisition times for SAX and LAX 2D LGE planes were obtained from the time stamps on the first and last images of the stack.

### Three-dimensional water-fat iNAV LGE sequence

The 3D inversion recovery prepared spoiled gradient-echo prototype sequence was performed in free breathing, covering the left ventricle in transverse orientation with the following parameters (Table 1): isotropic resolution of (1.3 mm)^3^, TR of 7.2 ms, TE1/TE2 of 2.38 ms/4.76 ms, bipolar gradient readout, receiver bandwidth of 496 Hz/px for both echoes, flip angle of 20°, FOV of 312 × 312 mm, matrix of 240 × 240, 30% phase oversampling (phase was right–left), 90% slice resolution, reconstructed to (1.3 mm)^3^, and number of averages of 1.

**Table 1:** Overview of the scan parameters for 2D and 3D LGE

	2D LGE	3D LGE
TR	8.35 ms	7.2 ms
TE	3.23 ms	TE1/TE2 = 2.38/4.76 ms
Flip angle	25°	20°
FOV	276 × 340 mm	312 × 312 mm
Matrix	156 × 256	240 × 240
Resolution	In-plane resolution 1.4 × 1.4 mm Slice thickness 8 mm, gap 2 mm	(1.3 mm)^3^ isotropic resolution

The inversion time was determined by an inversion time Look-Locker scout triggered on every heartbeat and ranged around 245 ± 28 ms.

The dual-echo 3D data were acquired with an undersampled variable-density golden step Cartesian trajectory with spiral profile order sampling (VD-CASPR) [24]. The actual acceleration factor was 2.6 compared to the fully sampled *k*-space. Low-resolution, coronal 2D dual-echo iNAVs were acquired immediately before the 3D acquisition, and the opposed-echo iNAV was used for motion estimation by tracking a manually selected template covering the whole heart along the left–right (LR) direction and the base and the mid part of the heart along the foot–head (HF) direction [25, 26]. Motion estimates in FH and LR directions were then used to correct the dual-echo 3D data to a reference position by modulating the *k*-space data with a linear shift [12]. After data acquisition was finished, the motion-corrected undersampled 3D data of both echoes were independently reconstructed with a compressed sensing (CS) reconstruction based on 3D regularization using orthogonal Haar wavelets [27]. The cost function of the CS reconstruction was solved with a fast iterative shrinkage-thresholding algorithm (FISTA) [28], and the proximal operator was weighted with the regularization parameter, which was set to 0.008. In all cases, the optimization was terminated after 20 iterations. Finally, water-fat separation was performed to obtain the final 3D LGE images [20]. All reconstructions were performed automatically in-line.

While scanning, the influence of the respiratory cycle on the image navigator was monitored in each patient.

### Image quality analysis

Two board-certified reviewers (M.Z. and W.W., 8 years and 10 years of experience in cardiovascular imaging, respectively) analyzed the images blinded to all patient and other imaging data. Three-dimensional LGE image datasets (water-only and in-phase images) were reformatted to short-axis planes to correspond with the 2D LGE slices and presented randomly using dedicated software (*syngo*.via; Siemens Healthcare). The highest possible resolution of 1.3 mm was used in order to fully benefit from the high-resolution sequence.

Overall image quality and artifacts not related to fat suppression were rated on a 5-point scale for 2D and 3D datasets [29]: 5 = excellent image quality, interpretable with no artifacts; 4 = good image quality, interpretable with minimal artifacts; 3 = average image quality, interpretation mildly degraded by image artifacts; 2 = below average image quality, interpretable but moderately degraded; and 1 = poor image quality, uninterpretable images. If present, the reason for impaired image quality was evaluated.

### Fat suppression in 3D water-fat LGE

Water-fat separation in the 3D LGE water-only images was evaluated using a 5-point scale [29]: 5 = excellent fat suppression quality, interpretable with no artifacts; 4 = good fat suppression, interpretable with minimal artifacts; 3 = average fat suppression, interpretation mildly degraded by image artifacts; 2 = below average image fat suppression, interpretable but moderately degraded; and 1 = poor fat suppression, uninterpretable images.

### LGE pattern and extent

Scar tissue was defined as visually hyperenhanced myocardium in the LGE sequence compared to remote myocardium. LGE characteristics were rated as ischemic when LGE involved the subendocardium in a coronary distribution. LGE was further characterized as subepicardial, patchy, midmyocardial, diffuse, involving RV insertion points, or pericardial. For segmentation, the 17-segment model was used as proposed by the American Heart Association (AHA) [30] and using the manual volume quantification tool in *syngo*.via. If present, the transmural LGE extent was estimated based on a 6-point scale [10]: 0 = 0%, 1 = 1–24%, 2 = 25–49%, 3 = 50–74%, 4 = 75–100%, 5 = striae, and 6 = diffuse. Enhancement of the pericardium was quantified according to a modified AHA 17-segment model. The right ventricular pericardium was divided into 6 segments, 2 per slice (basal, mid, apical). Diagnostic confidence for the presence of fibrosis/scar/pericarditis was assessed on a 3-point scale: 2 = LGE absent/present with confidence, 1 = probable LGE, and 0 = inconclusive.

### Statistical analysis

**Fig. 1:**
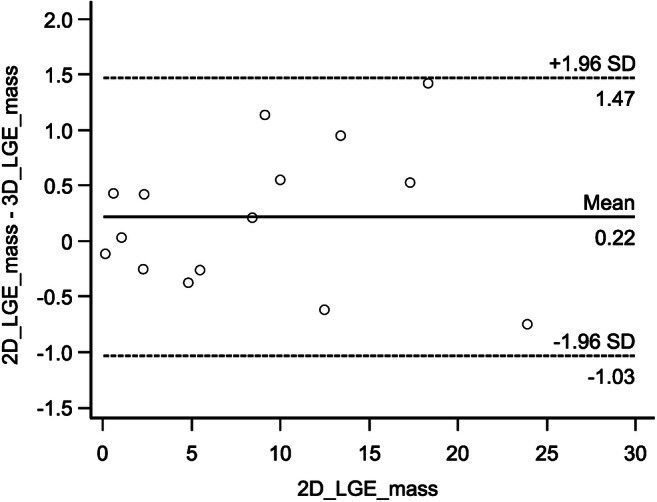
Pairwise comparison of myocardial 2D and 3D LGE volume size using the Bland–Altman plot. Δmass (2D_LGE_mass minus 3D_LGE_mass) is shown on the ordinate. 2D_LGE_mass is shown on the abscissa. Indicated are the limits of agreement and the mean difference. There were no outliers beyond the limits of agreement (± 1.96 × SD)

Interval-level data was evaluated for normal distribution using the Shapiro–Wilk test. In case of normal distribution, values are given as mean ± standard distribution (SD), otherwise and in case of ordinal-level data values given as median and range. Except for LGE volume quantification, the comparison between 3D and 2D LGE datasets was performed using the non-parametric Wilcoxon rank sum test. LGE volume quantification was evaluated using a paired *t* test and a Bland–Altman plot (Fig. 1). Significance was accepted for *p* values < 0.05.

Inter-reader agreement was evaluated by using Cohen’s kappa value (*κ*). *κ* was interpreted as follows: 0 < *κ* ≤ 0.2, slight agreement; 0.2 < *κ* ≤ 0.4, fair agreement; 0.4 < *κ* ≤ 0.6, moderate agreement; 0.6 < *κ* ≤ 0.8, substantial agreement; 0.8 < *κ* ≤ 1.0, almost perfect agreement; and *κ* = 1, perfect agreement. Statistical analysis was performed using the software package SPSS Statistics, version 21 (SPSS Inc./IBM). The Bland–Altman plot was performed using SciStat (MedCalc Software, Ltd).

## Results

The study population consisted of 24 men and 16 women (mean age 50.0 ± 15.4 years). Mean body mass index was 26.3 ± 5.0 kg/m^2^, and mean body surface area was 2.0 ± 0.3 m^2^. Mean heart rate was 69.5 ± 19.2/min. All patients had sinus rhythm. Occasional extrasystoles occurred in 4/40 patients. Mean LV ejection function was 53 ± 12%, mean LV end-diastolic volume (EDV) was 182 ± 69 ml (91 ± 29 ml/m^2^), and mean LV mass was 152 ± 51 g (74 ± 21 g/m^2^).

### Image quality

Both 2D LGE and 3D LGE delivered overall image quality scores of at least 3 in all cases. Image quality for LGE was comparable between 2D and 3D (water-only and in-phase) irrespective of the acquisition time point (3D acquisition prior to 2D: *p* = 0.44, 2D acquisition prior to 3D: *p* = 0.71, all 3D datasets compared to 2D: *p* = 0.67).

In 20/40 (50%) 2D LGE datasets, respiratory motion occurred. In 3D LGE (water-only and in-phase), motion artifacts were detected in 20/40 (50%) patients and insufficient myocardial nulling in 2/40 (5%, both in group 2). Inter-reader agreement was almost perfect (3D: *κ* = 0.84, 2D: *κ* = 0.92). See Table 2 for detailed image quality scores.

**Table 2:** Image quality scores for 2D and 3D LGE imaging

	5, *n* (%)	4, *n* (%)	3, *n* (%)	2 (%)	1 (%)
3D water-only (*n* = 40)	21 (53)	13 (33)	6 (15)	0	0
3D in-phase (*n* = 40)	21 (53)	13 (33)	6 (15)	0	0
2D (*n* = 40)	25 (63)	11 (28)	4 (10)	0	0

5 = excellent image quality, interpretable with no artifacts; 4 = good image quality, interpretable with minimal artifacts; 3 = average image quality, interpretation mildly degraded by image artifacts; 2 = below average image quality, interpretable but moderately degraded; 1 = poor image quality, uninterpretable images

### Fat suppression in water-only 3D Dixon LGE

Good or excellent fat suppression (scores > 3) was achieved in 37/40 (93%) cases. In 33/40 (83%) cases, fat suppression was excellent with no artifacts. In 4/40 (10%) cases, fat suppression was good with minimal artifacts, and in 3/40 (7%) cases, fat suppression was average due to sternal cerclages (1/40) and mild field inhomogeneity (2/40).

### LGE pattern and extent

In both 2D and 3D LGE (water-only and in-phase) imaging, LGE was rated as ischemic in 9 patients and non-ischemic in 11 patients while it was absent in 20 patients. LGE extent with respect to segmental or transmural myocardial enhancement was identical between 2D and 3D (water-only and in-phase) images. LGE size showed no significant difference (3D: mean 8.4 ± 7.2 g, 2D: mean 8.7 ± 7.3 g, *p* = 0.19). There were no outliers beyond the limits of agreement (± 1.96 × SD, Fig. 1). The level of confidence was comparable between both techniques (*p* = 0.66) with high confidence (score = 2) in 35/40 (87.5%) datasets for 2D and 3D (water-only and in-phase) imaging. All other cases were probable (score = 1), and no case was rated as inconclusive (score = 0) in both sequences.

Six of 11 (55%) patients with LGE presented with pericardial enhancement in both sequences. The 3D sequence detected more pericardial LGE especially along the right ventricle compared to 2D (3D water-only 88 segments [mean 14.7 ± 5.7 segments] vs. 3D in-phase 79 segments [mean 13.2 ± 5.2 segments] vs. 2D mean 69 segments [mean 11.5 ± 5.4 segments]).

**Fig. 2:**
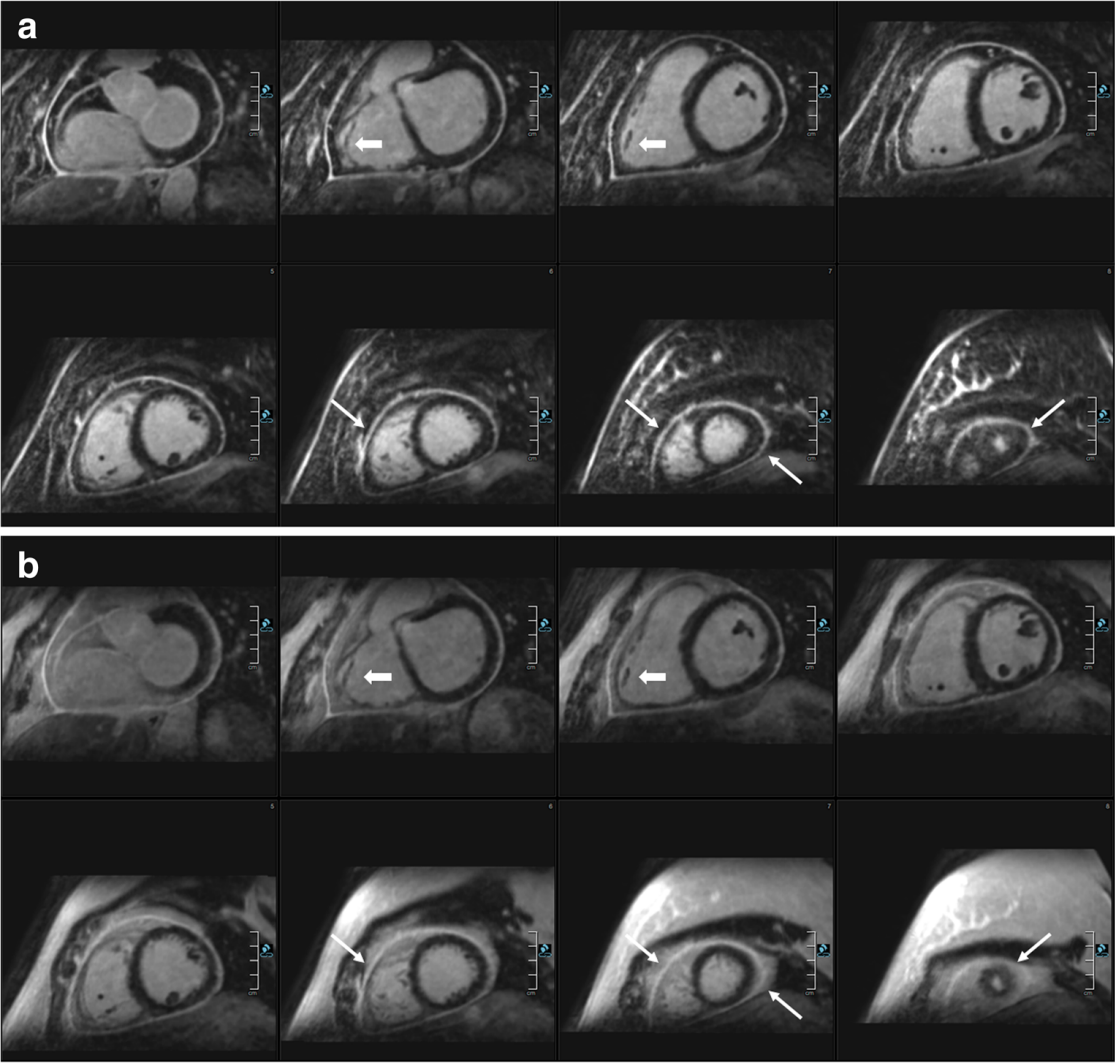

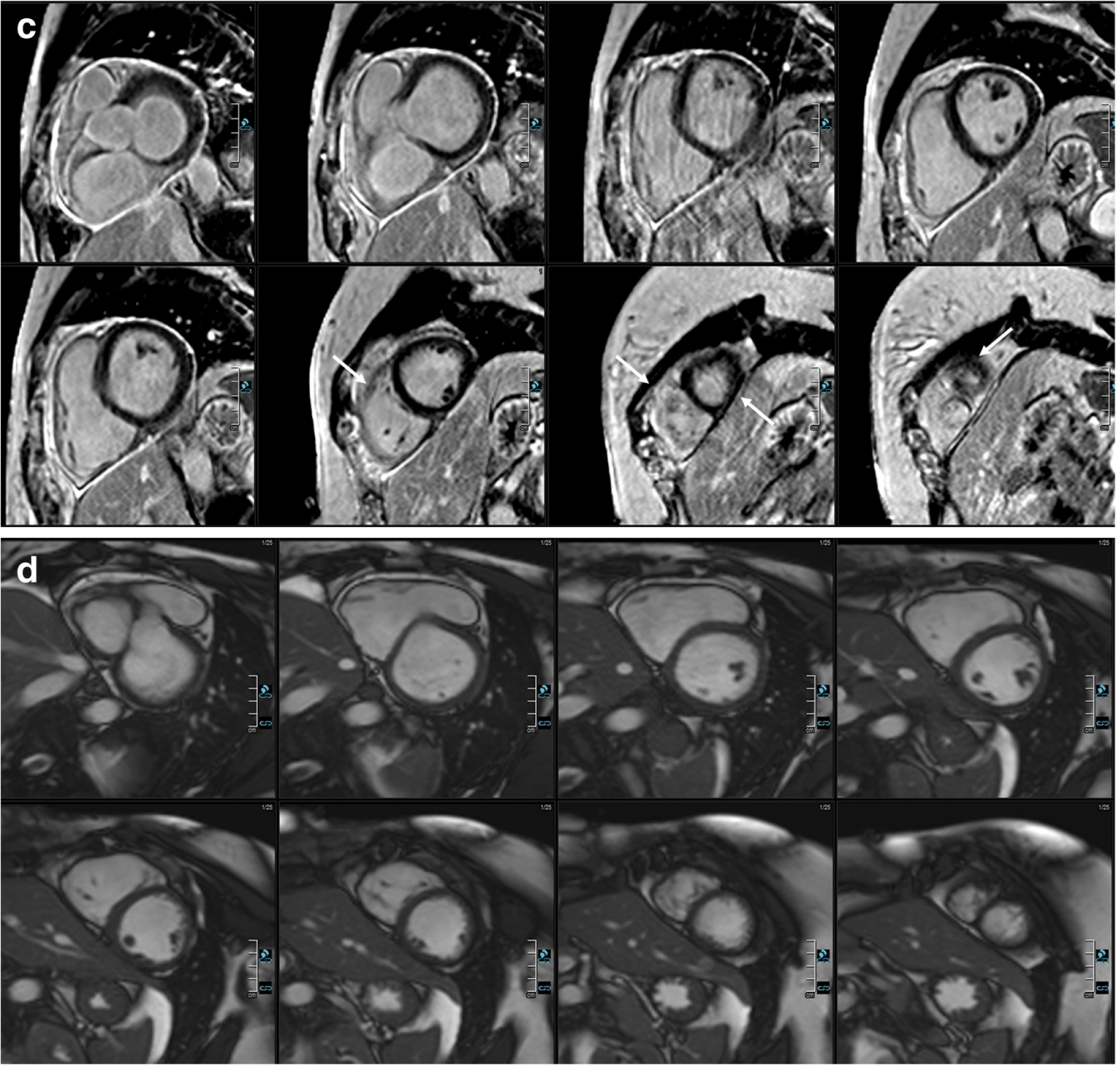
Comparison of 3D and 2D imaging in a female patient with pericarditis. Comparable short-axis reformatting of 3D water-only LGE images (**a**), 3D in-phase images (**b**), 2D PSIR LGE images (**c**), and bSSFP cine short-axis (SAX) images (**d**). Dixon-based fat suppression enables excellent delimitation of the enhanced pericardium against the epicardial fat. In direct comparison, the pericardium can hardly be identified in several areas in the 3D in-phase views and worse in the 2D PSIR LGE views (thin arrows: e.g., along the right ventricle, close to the apex). Moreover, 3D water LGE imaging allows for excellent depiction of small details such as the trabeculae of the right ventricle (bold arrows)

**Fig. 3:**
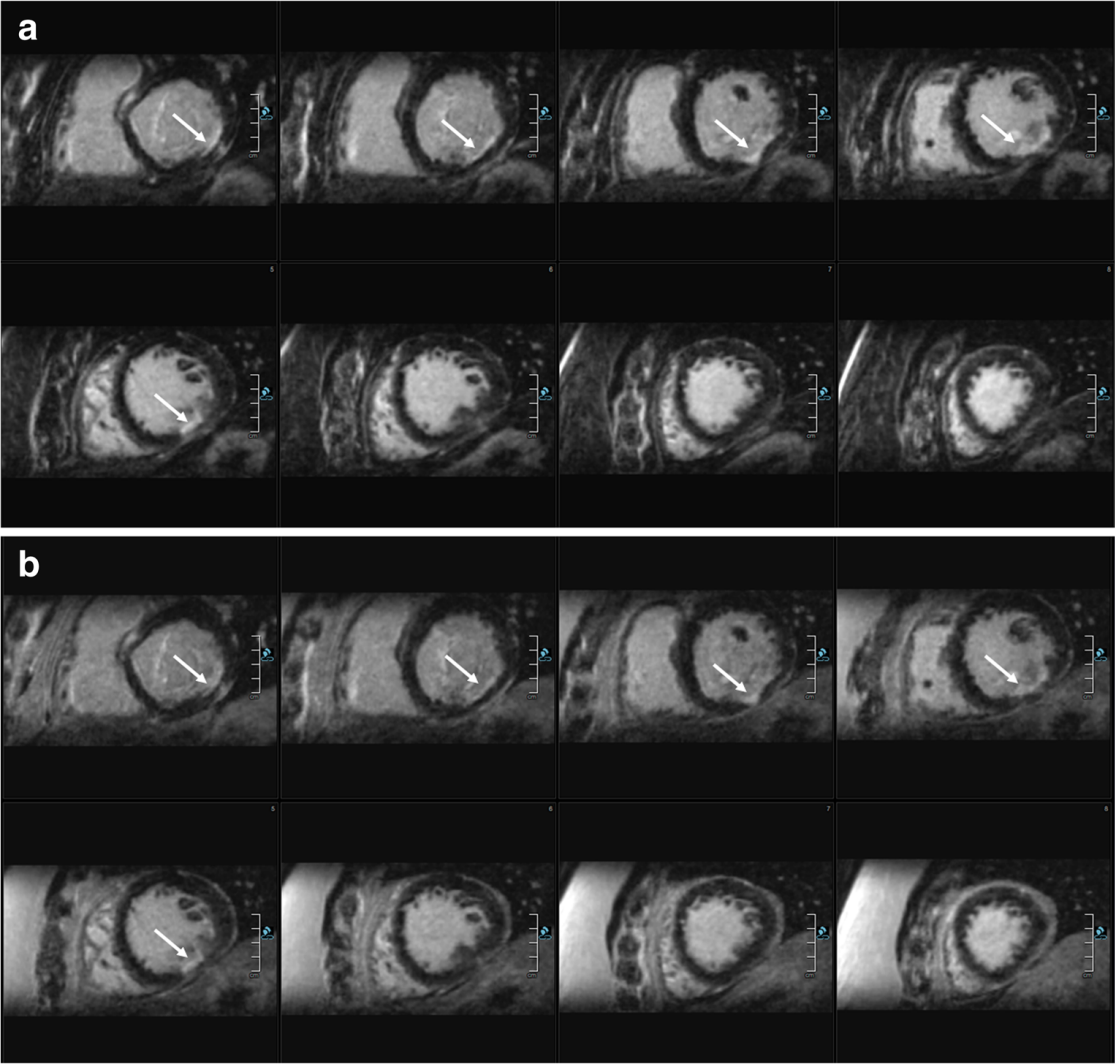

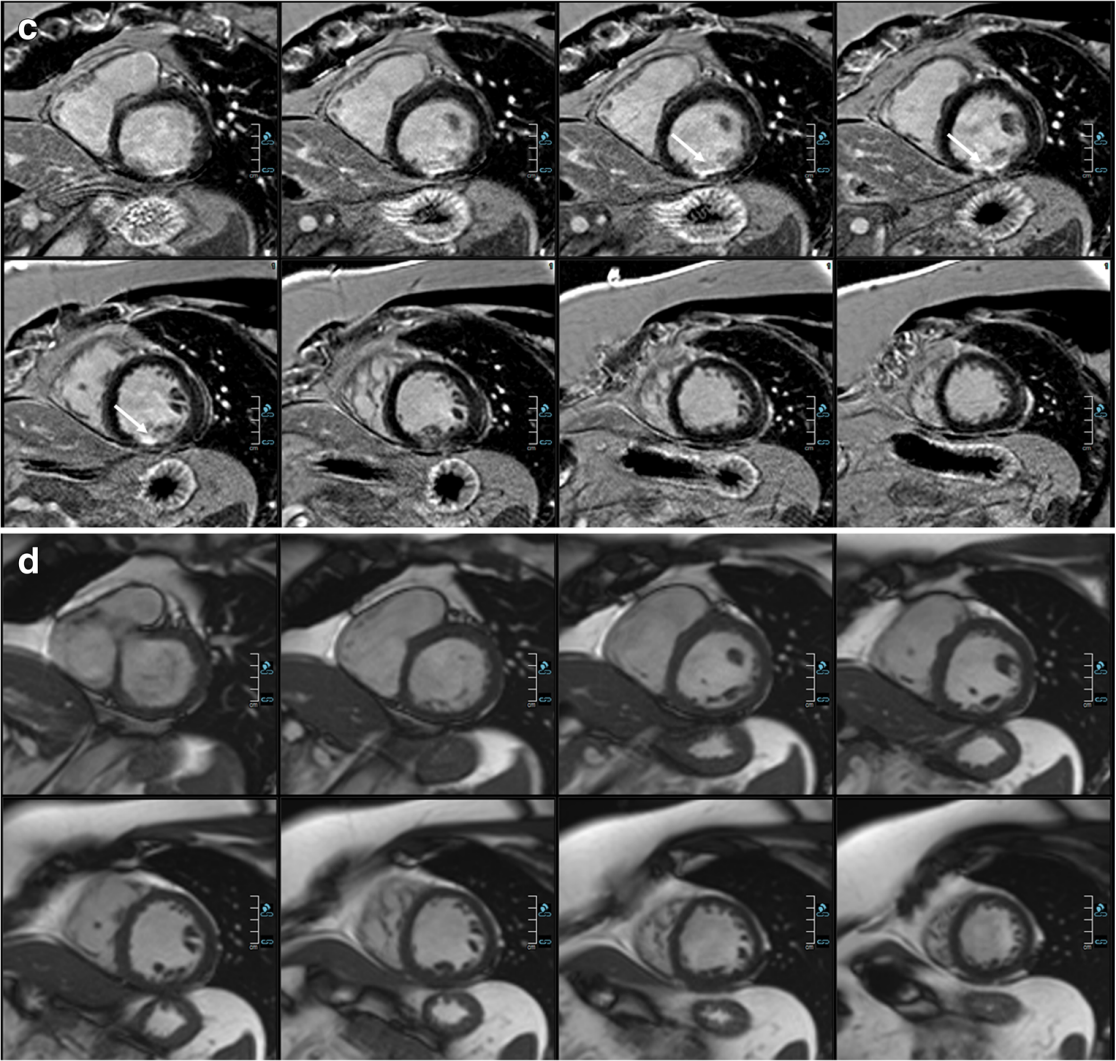
Comparison of 3D and 2D imaging in a male patient with myocardial infarction. Comparable short axis reformatting of 3D water-only LGE images (**a**), 3D in-phase images (**b**), 2D PSIR LGE images (**c**), and bSSFP cine SA images (**d**). Identical extent of LGE in the posterolateral basal and midventricular segments (arrows)

**Fig. 4:**
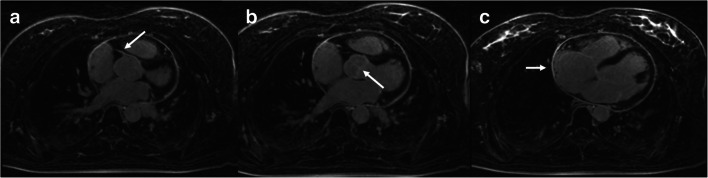
Three-dimensional water-only LGE imaging of a patient with pericarditis. Three-dimensional water-only LGE image with globally enhanced pericardium and homogenous fat suppression. Excellent depiction of smaller details such as the ostium of the right coronary artery (**a**), the aortic valve (**b**), and the right atrial wall (**c**) (arrows)

**Fig. 5:**
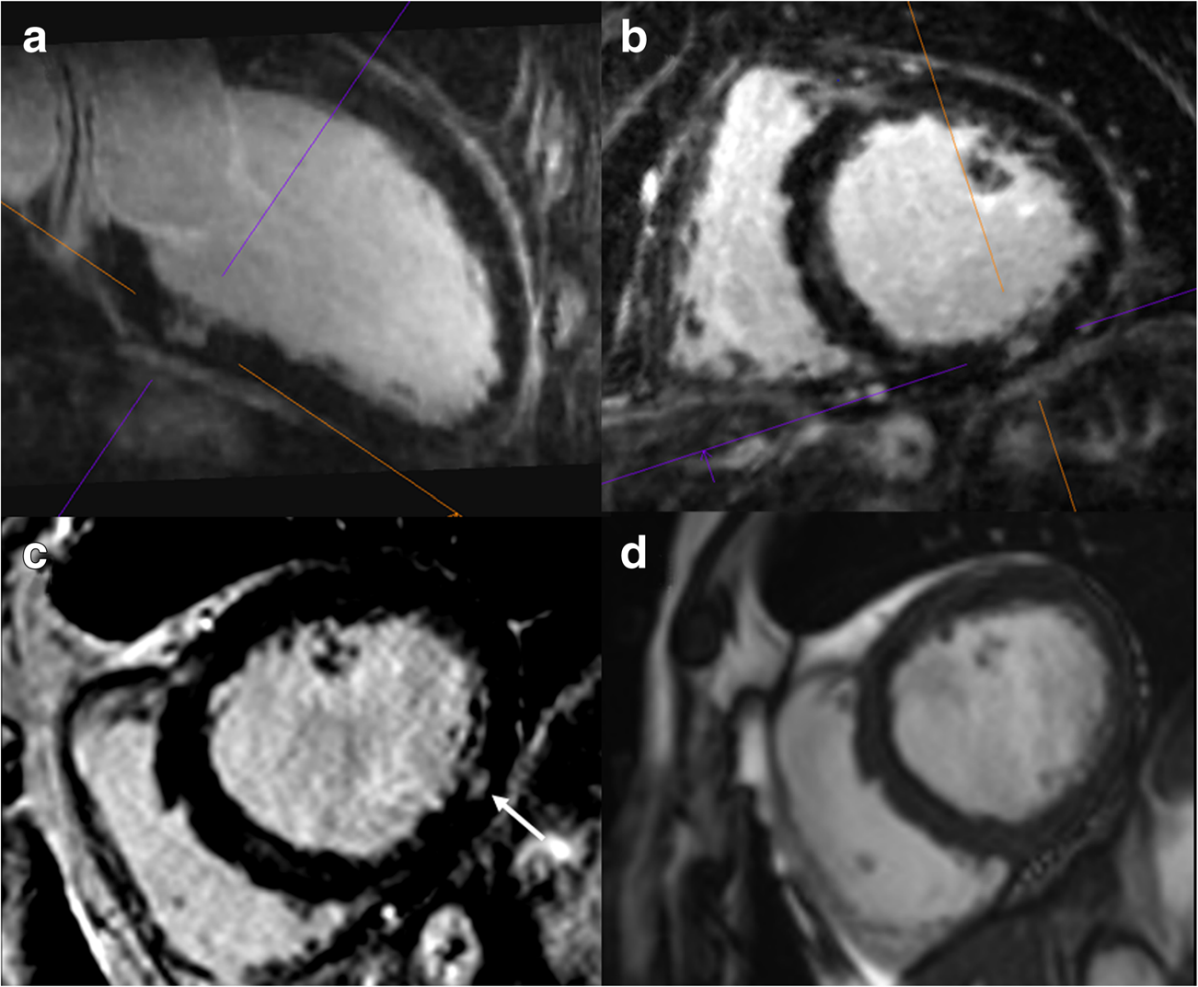
Comparison of 3D water-only (**a**, **b**) and 2D LGE PSIR (**c**) images in a patient with myocarditis. VLA: vertical long axis (2 chamber view), a small area of midmyocardial LGE in both the 3D (water-only, cross hairs) and 2D PSIR LGE (on the right, arrow) images. The isotropic high-resolution permits a 3D characterization with personalized views (views **a** and **b**, according to cross hairs). In the standard 2D LGE imaging protocol without additional views, this was the only slice depicting the small LGE. The corresponding SA cine image (**d**) excludes a myocardial crypt

Inter-reader agreement was almost perfect (2D *κ* = 0.88 and 3D *κ* = 0.90). See Tables 3 and 4 for detailed LGE pattern and extent evaluation and Figs. 2, 3, 4, and 5 for examples.

**Table 3:** LGE pattern for 2D and 3D LGE imaging

	0, *n* (%)	1, *n* (%)	2, *n* (%)	3, *n* (%)	4, *n* (%)	5, *n* (%)	6, *n* (%)
3D water-only (*n* = 40)	20 (50)	9 (23)	4 (10)	0 (0)	2 (5)	0 (0)	6 (15)
3D in-phase (*n* = 40)	20 (50)	9 (23)	4 (10)	0 (0)	2 (5)	0 (0)	6 (15)
2D (*n* = 40)	20 (50)	9 (23)	4 (10)	0 (0)	2 (5)	0 (0)	6 (15)

0 = no LGE, 1 = ischemic, 2 = patchy, 3 = subepicardial, 4 = mid wall, 5 = RV insertion points, 6 = pericardial

**Table 4:** LGE transmural extent for 2D and 3D LGE imaging

	Total	1, *n* (%)	2, *n* (%)	3, *n* (%)	4, *n* (%)	5, *n* (%)	6, *n* (%)	7, *n* (%)
3D water-only	142	0 (0)	18 (13)	10 (7)	24 (17)	2 (1)	0 (0)	88 (62)
3D in-phase	133	0 (0)	18 (14)	10 (8)	24 (18)	2 (2)	0 (0)	79 (59)
2D	123	0 (0)	18 (15)	10 (8)	24 (20)	2 (2)	0 (0)	69 (56)

1 = 1–25%, 2 = 26–50%, 3 = 51–75%, 4 = 76–100%, 5 = striae, 6 = diffuse, 7 = pericardium

### Sequence duration

Scan duration was ~ 10 min for both 2D and 3D LGE (water-only and in-phase) acquisitions, although it was significantly shorter for 2D compared to 3D (2D median 9:32 min [range 5:09–20:15 min, interquartile range (IQR) 3:00 min] vs. 3D median 10:46 min [range 7:42–15:50 min, IQR 2:00 min], *p* = 0.001). The 2D acquisition required typically between 13 and 15 breath-holds, whereas 3D LGE was performed under free breathing. The respiratory pattern and depth had no impact on the image navigator. Besides very few exceptions (non-respiratory-dependent patient movement), the navigator depicted the heart reliably.

Occasional arrhythmias prolonged the scan time due to skipped heart beats. Due to the small number of patients (4/40), this was not statistically evaluable. The pauses between breath-holds were accounted in the scan time in 2D LGE imaging. The resting time between breath-holds was approximately 10 s plus a respiratory command (approx. 7 s: “breath in–breath out”) for all patients.

### Post-contrast delay

There was no significant difference in post-contrast delay between 2D and 3D LGE images (2D mean 23:21 ± 9:32 min, 3D mean 19:29 ± 6:23 min, *p* = 0.17).

## Discussion

In this study, we demonstrated the feasibility of a free-breathing, image-navigated isotropic high-resolution 3D LGE sequence with compressed sensing and Dixon water-fat separation in a routine clinical setting. Detection of LGE, extent, and size was comparable between 3D (Dixon fat suppression and in-phase images) and our standard 2D images without fat suppression. Overall image quality was comparable between both 3D (water-only and in-phase) and 2D sequences delivering diagnostic image quality in all cases. The Dixon fat suppression enabled a better visualization of pericardial enhancement in pericarditis due to good/excellent fat suppression.

One of the major challenges in MRI is motion, and especially in conventional cardiac imaging, image quality is often degraded by motion artifacts. Three-dimensional acquisitions are even more susceptible to motion artifacts due to long scan times. In order to overcome this problem, free-breathing acquisitions using diaphragmatic navigator gating have been proposed for 3D water-fat LGE imaging [21, 31]. Previously published studies with high resolution suffered of prolonged scan times, e.g., around 16 ± 7.19 min at 1.4 mm isotropic resolution [32]. These were performed with radial imaging [11] or were restricted to dedicated areas of the heart [5]. In our study, the free-breathing 3D scan duration was on average only 74 s longer than conventional breath-held 2D imaging, with the former covering all cardiac structures including the atria with an isotropic resolution of 1.3 mm. In contrast to standard 2D imaging, the 3D approach allows for individual multiplanar reformatting without the need of additional acquisitions to visualize small areas of LGE as demonstrated in Fig. 5. Furthermore, since 3D measurements are performed every heartbeat with an iNAV-based motion correction approach enabling 100% respiratory scan efficiency (no data rejection), the duration of the 3D sequence is known a priori with high reliability as demonstrated by Bratis et al. [10] Therefore, the scan duration depends only on the patient’s heart rate and duration of the resting phase of the cardiac cycle and is not altered by changes in the respiratory pattern. The relatively wide scan duration range in both 2D LGE and 3D LGE is due to the wide range of heart rates and skipped heart beats in intermittent arrhythmias. With predictable scan durations, inline reconstruction, and rapidly available images, the evaluated 3D approach is easily implementable in clinical routine.

High-resolution coverage of the entire heart allows for accurate detection of myocardial scar in ischemic heart disease which is an important predictor of functional recovery after revascularization [33]. In a previously published study evaluating the iNAV technique without compressed sensing and fat suppression, the excellent agreement between 2D and 3D datasets with regard to global, segmental LGE detection and transmurality was reported [10]. This is in line with our results as LGE extent and pattern were comparable between conventional 2D and water-fat 3D imaging. However, in almost 50% of the 3D datasets, motion artifacts occurred, leading to at least slightly impaired image quality. This might be explained by the fact that the iNAV-based motion compensation was, in our implementation, restricted to rigid foot–head and left–right motion of the heart, and therefore, non-rigid deformations of the heart due to breathing were not corrected for. Non-rigid motion-compensated reconstructions may further improve image quality as demonstrated for similar 3D imaging approaches initially developed for coronary MR angiography [34, 35]. With improved motion correction, the 3D sequence with an isotropic 1.3 mm resolution as evaluated in our study has the potential to offer significant advantages especially in thinner structures such as the atria or the right ventricle.

Besides spatial resolution and scan time, fat suppression has the potential to improve diagnostic accuracy of cardiac MRI. The differentiation of fat and embedded structures like the pericardium is critical in clinical routine. In the small patient subgroup with pericarditis, the water-only images of the Dixon technique seem to be beneficial for the detection of pericardial involvement due to a better discrimination of epicardial fat and contrast-enhanced pericardium. The Dixon technique provided robust water-fat separation in all cases and improved visualization of the enhanced pericardium in patients with pericarditis especially along the right ventricular free wall surrounded by epicardial fat. The option to evaluate in-phase and water-only images is not only a promising tool for patients with pericarditis, but it could also be used for detection of fibrofatty infiltration of the right ventricle in ARVD [20].

A challenge in segmented 3D LGE acquisitions is the potential change of inversion time due to contrast washout during the scan, potentially leading to less optimal blood/myocardium contrast compared to conventional 2D images [32]. Dynamic inversion time has therefore been proposed [36]. This being said, in our observation, insufficient myocardial nulling was a minor concern and occurred in only two patients. Furthermore, there was no significant difference in image quality between 2D and 3D LGE imaging irrespective of the acquisition time point (groups 1 and 2).

### Limitations

First, the sample size was small and patients with permanent arrhythmia or critically ill patients with limited breath-hold capacity were not included. Studies including this patient cohort should be performed in the future to evaluate the performance and the image quality of the Dixon sequence in more ill patients.

Second, we compared the 3D sequence to our institutional standard 2D sequence without fat suppression according to the current guideline for standardized CMR protocols [23]. Due to time constraints, a second 2D LGE sequence with fat suppression was not evaluated.

Third, additional quantitative parameters like signal-to-noise ratio (SNR) or contrast-to-noise ratio (CNR) were not assessed in our study as there is no commonly accepted gold standard for quality assessment of non-linear reconstruction algorithms such as compressed sensing because SNR cannot be reliably computed [37].

## Conclusion

Free-breathing, isotropic high-resolution 3D LGE imaging with image navigation, undersampled spiral-like Cartesian sampling, compressed sensing reconstruction, and Dixon water-fat separation yields comparable LGE detection compared to a conventional 2D approach. The slightly longer scan times come with complete cardiac coverage, higher spatial resolution, and the option of multiplanar reformations. With one scan, the technique provides fat-suppressed and non-fat-suppressed images omitting the need for an additional scan. The robust fat suppression seems to be especially beneficial in patients with pericarditis. Non-rigid motion correction will most likely improve 3D image quality further, which will ease its implementation into clinical routine protocols.

## Abbreviations

ARVD: Arrhythmogenic right ventricular dysplasia
bSSFP: Balanced steady-state free precession
CMR: Cardiac magnetic resonance imaging
iNAV: Image navigation
LGE: Late gadolinium enhancement
MRI: Magnetic resonance imaging
PSIR: Phase-sensitive inversion recovery
TE: Echo time
TR: Repetition time
